# Prediction of *trans*-antisense transcripts in *Arabidopsis thaliana*

**DOI:** 10.1186/gb-2006-7-10-r92

**Published:** 2006-10-13

**Authors:** Huan Wang, Nam-Hai Chua, Xiu-Jie Wang

**Affiliations:** 1 State Key Laboratory of Plant Genomics, Institute of Genetics and Developmental Biology, Chinese Academy of Sciences, Beijing 100101, China; 2 Graduate University of the Chinese Academy of Sciences, Beijing 100101, China; 3 Laboratory of Plant Molecular Biology, The Rockefeller University, New York, NY 10021, USA

## Abstract

**Background:**

Natural antisense transcripts (NATs) are coding or non-coding RNAs with sequence complementarity to other transcripts (sense transcripts). These RNAs could potentially regulate the expression of their sense partner(s) at either the transcriptional or post-transcriptional level. Experimental and computational methods have demonstrated the widespread occurrence of NATs in eukaryotes. However, most previous studies only focused on *cis*-NATs with little attention being paid to NATs that originate in *trans*.

**Results:**

We have performed a genome-wide screen of *trans*-NATs in *Arabidopsis thaliana *and identified 1,320 putative *trans*-NAT pairs. An RNA annealing program predicted that most *trans*-NATs could form extended double-stranded RNA duplexes with their sense partners. Among *trans*-NATs with available expression data, more than 85% were found in the same tissue as their sense partners; of these, 67% were found in the same cell as their sense partners at comparable expression levels. For about 60% of *Arabidopsis trans*-NATs, orthologs of at least one transcript of the pair also had *trans*-NAT partners in either *Populus trichocarpa *or *Oryza sativa*. The observation that 430 transcripts had both putative *cis*- and *trans*-NATs implicates multiple regulations by antisense transcripts. The potential roles of *trans*-NATs in inducing post-transcriptional gene silencing and in regulating alternative splicing were also examined.

**Conclusion:**

The *Arabidopsis *transcriptome contains a fairly large number of *trans*-NATs, whose possible functions include silencing of the corresponding sense transcripts or altering their splicing patterns. The interlaced relationships observed in some *cis*- and *trans*-NAT pairs suggest that antisense transcripts could be involved in complex regulatory networks in eukaryotes.

## Abstract 2

A genome-wide screen for trans-natural antisense transcripts in *Arabidopsis thaliana *suggests that antisense transcripts could be involved in complex regulatory networks in eukaryotes.

## Background

Natural antisense transcripts (NATs) are endogenous RNA molecules with sequence complementarity to other RNAs (sense transcripts). Depending on their genomic origins, natural antisense transcripts can be classified into two groups, *cis*-NATs and *trans*-NATs. *Cis*-NATs are transcripts derived from the same genomic loci as their sense counterparts, but from different chromosome strands, whereas *trans*-NATs and their sense partners originate from distinct genomic regions. Genes encoding *cis*-NATs resemble overlapping open reading frames (ORFs) commonly seen in prokaryotes and viruses, but such overlapping genes were thought to be rare in eukaryotes [1]. Recent research advances in eukaryotic natural antisense transcripts, however, have challenged this view. Genome-wide computational and experimental studies have shown that about 5% to 10% of gene transcripts in mammals and plants have *cis*-NATs, whilst information on *trans*-NATs is still not yet available [1-7].

Emerging lines of evidence have shown that NATs play important roles in the regulation of many gene expression related processes, such as transcriptional exclusion, RNA interference, alternative splicing, DNA methylation, RNA editing and X-chromosome inactivation [8-17]. Antisense transcripts have been shown to regulate expression of the mouse *Msx1 *gene, which encodes a homeobox transcription factor controlling craniofacial development [18]. Malfunction of antisense transcripts are known to cause some human diseases, such as cancer (reviewed in [19]). Widespread antisense regulations have also been detected in plants, with the identification of 687 *cis*-NAT pairs in rice and more than 1,000 pairs in *Arabidopsis *[5-7]. Phylogenetic analysis has revealed that the positions and overlapping patterns of genes producing *cis*-NAT pairs tend to be more conserved during evolution than unrelated genes in vertebrates, indicating the functional importance of antisense regulation [20].

Most studies on antisense transcripts have so far focused only on NATs of *cis*-origins because their relationships are easier to identify. However, as a major member of the antisense transcript family, *trans*-NATs also widely exist and seem to have important functions. In an attempt to search for mammalian NATs using experimental approaches, Rosok and Sioud [21] reported that about 50% of the cloned double-stranded RNAs in human normal mammary epithelial and breast cancer cells are *trans*-NATs. A systematic screening of NATs in several fungal genomes also uncovered many *trans*-NATs that could potentially participate in complex gene expression networks [22]. It should be noted that *trans*-NATs discussed here and in the remainder of this paper only refer to long transcripts that can form partial or complete complementary double-stranded RNA duplexes with other *trans*-originated long RNA transcripts. Several classes of small non-coding RNAs that also function in *trans*, such as microRNAs, small interfering (si)RNAs and small nucleolar RNAs, are not within the scope of this work.

We have previously used computational methods to identify *cis*-NATs in *Arabidopsis thaliana *[7]. To further understand gene expression networks regulated by antisense transcripts, we performed a genome-wide screen of *trans*-encoded NATs in *Arabidopsis *and identified 1,320 *trans*-NAT pairs. By inspecting the structure of putative RNA-RNA duplexes at the minimum hybridization energy, we confirmed the predicted antisense relationship of the majority of putative *trans*-NAT pairs *in silico*. Among *trans*-NATs with available expression data, more than 85% were found in the same tissue as their sense partners. A systemic screen of *in situ *hybridization data of *Arabidopsis *root cells showed that 67% of *trans*-NAT pairs with available data for both transcripts could be detected in the same root cells at comparable expression levels. The orthologs of at least one transcript of about 60% of *Arabidopsis trans*-NAT pairs also had *trans*-encoded antisense partners in poplar or rice, sometimes in both species. The potential gene expression regulatory networks formed by *cis*- and *trans*-NATs were analyzed using transcripts of UDP-glucosyl transferase family members as examples. We also explored the potential functions of *trans*-NATs in post-transcriptional gene silencing and in regulating alternative splicing.

## Results

### Prediction of *Arabidopsis trans*-NAT pairs

To identify *trans*-NATs in *Arabidopsis*, we first collected sequences of all *Arabidopsis *annotated genes and full-length cDNA transcripts, and grouped them into clusters according to their genomic locations. Here, a transcript cluster represented a group of all transcripts derived from the same gene or genomic locus. A genome-wide *trans*-NAT screen was carried out by searching for transcript cluster pairs sharing sequence complementarity with each other using the NCBI BLAST program. Two transcripts were considered as a *trans*-NAT pair if: they have partial or perfect sequence complementary regions that could form RNA-RNA duplexes; the total length of all putative duplex regions of the two transcripts is longer than 50% of the length of the shorter transcript of the pair (high-coverage category); or the length of the longest putative duplex region of the two transcripts is greater than 100 nucleotides (nt; 100 nt category). After removing previously reported *cis*-NATs and pairs formed by transcripts derived from annotated transposons and pseudogenes, a total of 1,320 *trans*-NAT pairs were identified within the *Arabidopsis *genome (Additional data file 1). Among them, 368 *trans*-NAT pairs belonged to the 'high-coverage' category, whilst the remaining 952 pairs were from the '100 nt' class (Table 1). The average length of the double-stranded pairing region of the 'high-coverage' class *trans*-NAT pairs is 571 nt, with a range between 75 and 2,628 nt. For the '100-nt' class *trans*-NAT pairs, the average pairing length is 258 nt, with a range between 100 and 1,621 nt.

**Figure 1:**
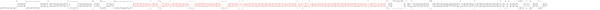
Annealed structure of a *trans*-NAT pair (At4g19270::At1g56530). The annealed structure of two transcripts was predicted by the hybrid program. Transcript At4g19270 is shown as the upper strand from 5' to 3', whilst transcript At1g56530 is shown as the lower strand from 3' to 5'. The paired region obtained by the blast search result is shown in red.

RNA molecules are known to assume various three-dimensional structures to execute their biological functions or to interact with other molecules. To investigate whether two transcripts of a putative *trans*-NAT pair could indeed form a double-stranded RNA duplex, we used a hybrid program [23,24] to inspect the melting structure of each *trans*-NAT pair *in silico*. The results show that the two transcripts of all predicted *trans*-NAT pairs in the high-coverage category and about 90% of the pairs in the 100 nt category could hybridize to each other and have extended duplex regions in their lowest energy melting forms, at least based on the *in silico *RNA hybridization model (see Materials and methods). Some *trans*-NAT pairs even had a double-stranded pairing region extending beyond the predicted area based on BLAST results (Figure 1).

### Expression analysis of *trans*-NATs

Among the 1,320 *trans*-NAT pairs, 658 pairs were formed by two transcript clusters both of which had matching full-length cDNAs, 444 pairs had full-length cDNA support for one transcript, and the remaining 218 pairs were identified solely by comparing annotated gene sequences (Table 1).

For an RNA molecule to function as *trans*-NAT, it has to co-exist with its sense transcript in the same cell in order to form double-stranded RNA duplex. To check the possibility of co-expression of the putative *trans*-NAT pairs, we used the *Arabidopsis *public MPSS database to examine the expression profiles of transcripts in different tissues or under different growth conditions. The *Arabidopsis *public MPSS database contains 17 nt and 20 nt long expressed sequence tags of *Arabidopsis *transcripts from 17 different tissues or plants grown under different conditions. In this study, we first mapped all 17 nt and 20 nt MPSS tags to the *Arabidopsis *genome, and selected for further analysis only those tags that could be uniquely mapped to transcripts forming *trans*-NAT pairs. About 16% of *trans*-NAT pairs in the 'high-coverage' category and 28% of *trans*-NAT pairs in the '100 nt' category had corresponding MPSS tags for both transcripts, and another 32% and 45% *trans*-NAT pairs in the 'high-coverage' and the '100 nt' categories, respectively, had MPSS tags for one transcript (Table 2). For those *trans*-NAT pairs in which both transcripts had matching MPSS data, more than 85% were co-expressed in at least one tissue (Table 2), suggesting that the two transcripts of these *trans*-NAT pairs had the opportunity to form double-stranded RNA duplexes *in vivo*. The expression patterns of two *trans*-NAT pairs derived from the MPSS data are shown in Table 3 as examples. We note that, in most cases, the sense and antisense transcripts of a *trans*-NAT pair had comparable expression levels when expressed in the same tissue. No significant tissue bias was observed in the expression of *trans*-NAT pairs when comparing MPSS data from the 17 different libraries.

To further investigate the potential of putative *trans*-NAT pairs to form double-stranded RNA duplexes at the single cell level, we inspected the expression pattern of each *trans*-NAT pair in *Arabidopsis *root cells using publicly available *in situ *hybridization data (AREX database) [25]. Since the AREX database contains information only for annotated *Arabidopsis *genes, only 658 putative *trans*-NAT pairs for which both transcripts derived from annotated genes could be compared by this analysis. Among the 355 *trans*-NAT pairs with *in situ *hybridization data for both transcripts, mRNAs of both transcripts of 237 pairs (67%) were found in the same cell with comparable expression levels (Table 4), suggesting that the sense and antisense transcripts of these pairs have the opportunity to interact with each other in *Arabidopsis *root cells. Whether sense and antisense transcripts in the same cell might be present in different cellular compartments awaits future experimental investigations. A complete list of the 355 *trans*-NAT pairs with available *in situ *hybridization data is provided in Additional data file 2.

### Functions of *trans*-NAT pairs

**Figure 2:**
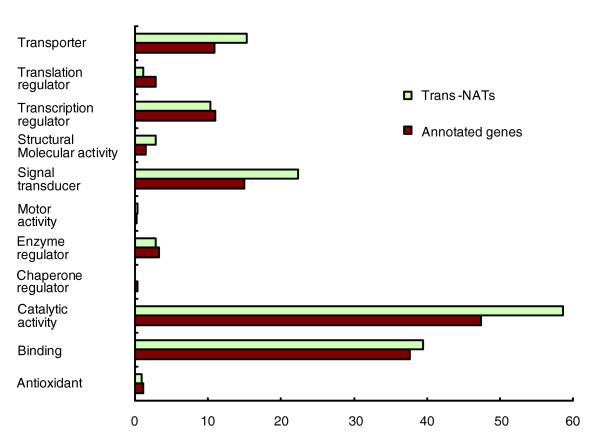
Functional analysis of *trans*-NATs using GO. The percent of *Arabidopsis *annotated genes and genes involved in *trans*-NAT pairs in each functional category are shown.

We used the *Arabidopsis *function assignment from the Gene Ontology (GO) consortium to analyze the biological functions of *trans*-NATs and observed a modest functional category bias. Transcripts from function classes with catalytic activity, signal transducer activity and transporter activity were slightly over-represented (Figure 2). Chi-square test results showed that the difference between transcripts of *trans*-NAT pairs verses those from the whole genome had a p value < 0.01 in all the above categories, indicating that the difference was statistically significant. A detailed gene function analysis using FuncAssociate [26] revealed that transcripts from several gene families or functional groups were over-represented in *trans*-NAT pairs, including transcripts of UDP-glycosyltransferase genes, and gene transcripts involved in cell wall biosynthesis, protein ubiquitination and responses to auxin stimulus (Table 5). By contrast, no enrichment in any specific gene family was found among transcripts of *cis*-NAT pairs (data not shown).

### Evolutionary conservation of *trans*-NAT pairs

To study the possible phylogenetic conservation of *trans*-NATs in higher plants, we performed an *in silico *search for *trans*-NAT pairs in poplar and rice and compared them with those from *Arabidopsis*. For about 60% of *Arabidopsis trans*-NAT pairs, homologs of at least one transcript involved in the pair also have *trans*-NAT partners in either poplar or rice (Table 6). For the majority of these *Arabidopsis trans*-NAT pairs, only one transcript retained a *trans*-NAT relationship in poplar or rice, but with new partners. Even for the small proportion of *Arabidopsis trans*-NAT pairs in which both transcripts retained *trans*-NAT relationships in poplar or rice, the sense and antisense transcripts of the same *trans*-NAT pair tended to have new pairing partners; only one *trans*-NAT pair remained the same in poplar and rice as in *Arabidopsis*.

### Networks formed by *cis*- and *trans*-NAT pairs

**Figure 3:**
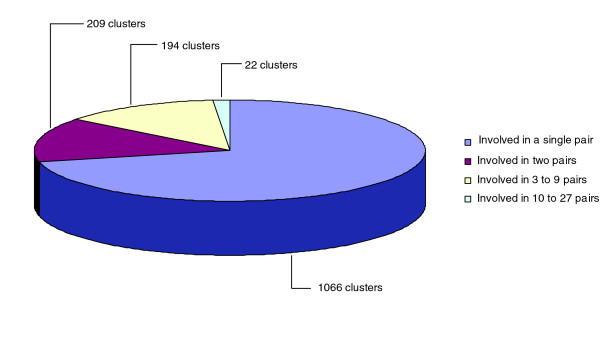
Pairing relationship of transcript clusters in *trans*-NAT pairs.

Unlike *cis*-NAT pairs, of which one sense transcript usually has only one antisense partner, one-to-many relationships are commonly seen in *trans*-NATs. There were also cases in which one transcript formed different double-stranded RNA duplexes with different transcripts derived from the same gene as a result of alternative splicing. Among all transcript clusters involved in *trans*-NAT pairs, 425 from both the high-coverage category and the 100 nt category can form multiple *trans*-NAT pairs with other transcripts (Figure 3).

**Figure 4:**
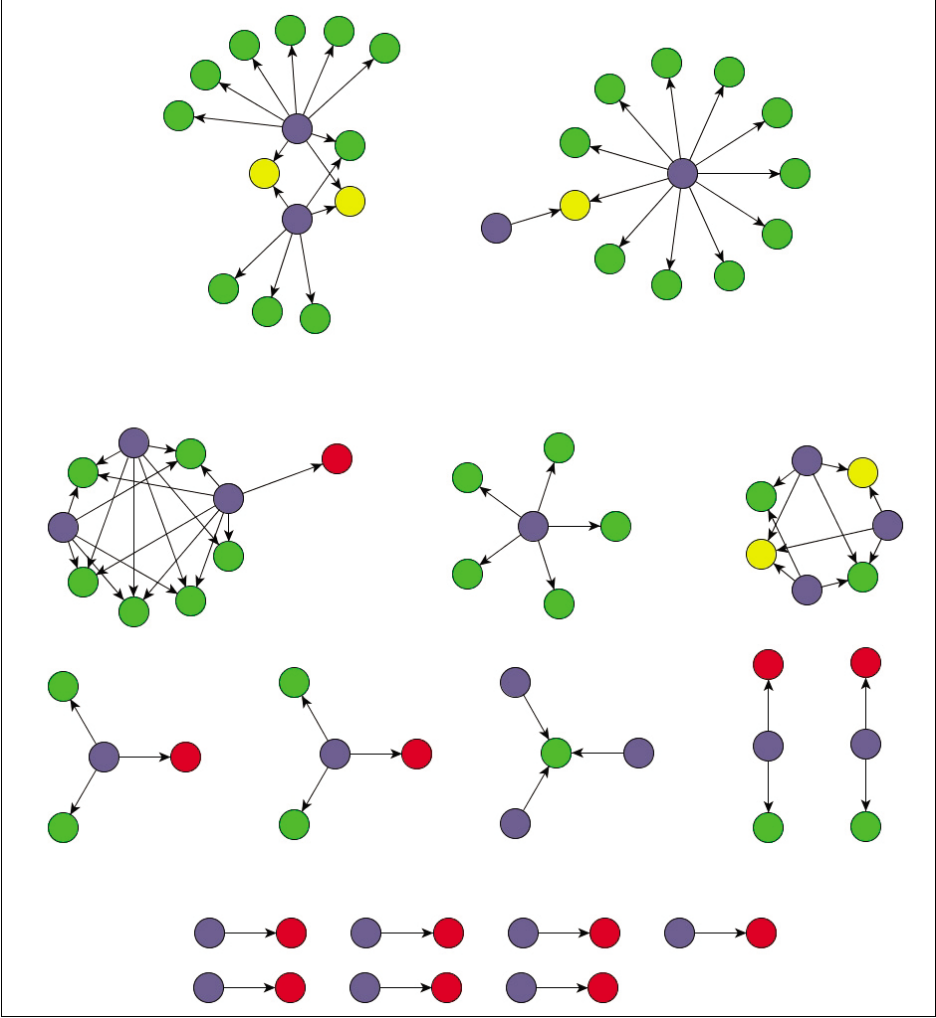
Networks of *cis*- and *trans*-NAT pairs formed by transcripts encoding UDP-glucosyl transferase family proteins in *A. thaliana*. Green ellipses represent UDP-glucosyl transferase transcripts involved in *trans*-NAT pairs only; red ellipses represent UDP-glucosyl transferase transcripts involved in *cis*-NAT pairs only; yellow ellipses represent UDP-glucosyl transferase trasncripts involved in both *cis*- and *trans*-NAT pairs. Transcripts from other protein families are shown as blue ellipses. Directed lines present the pairing relationship of two transcripts, with arrows pointing to UDP-glucosyl transferase transcripts.

Comparison with previously reported *Arabidopsis cis*-NAT data revealed that 430 transcripts on the *trans*-NAT list also had *cis*-NATs [7], indicating that antisense transcripts might form complex regulatory networks in *Arabidopsis*. UDP-glucosyl transferase family proteins are important enzymes catalyzing the transportation of sugars [27]. The *Arabidopsis *genome contains about 115 genes encoding UDP-glucosyl transferase family proteins. Transcripts of 44 UDP-glucosyl transferase genes have one or more pairing *trans*-NATs, among which 5 also have putative *cis*-NATs. Another 13 UDP glucosyl transferase gene member transcripts have pairing *cis*-NATs only. We analyzed NAT pairs formed by transcripts of UDP-glucosyl transferase gene family members in detail using the yEd software [28] to uncover possible regulatory networks formed by antisense transcripts (Figure 4). Our results showed that antisense transcripts could potentially regulate the UDP-glucosyl transferase family transcripts in various ways. Some transcripts could form antisense pairs with transcripts of UDP-glucosyl transferase family members in both a *cis*- and *trans*-manner. Phylogenetic analysis of UDP-glucosyl transferase gene member transcripts indicated that closely related transcripts (from the same clade of the phylogenetic tree) tended to be regulated by the same *trans*-antisense transcript (Figure 4, Additional data file 3). Such a complex pairing network was also observed amongst transcripts of several other gene families (data not shown).

### Potential roles of *trans*-NATs in inducing gene silencing

It has been shown that double-stranded RNA duplexes could be digested by Dicer to produce small interfering RNAs (reviewed in [29]). Since *trans*-NAT pairs also have long extended double-stranded regions, we asked whether some, if not all, of them could regulate each other's expression via the RNA interference pathway. To test this hypothesis, we first mapped all available *Arabidopsis *small RNAs from the public *Arabidopsis *MPSS database to the *Arabidopsis *genome [30], and searched for those siRNAs that could presumably be generated by *trans*-NAT pairs. We were able to identify a total of 148 siRNAs that were putatively derived from the RNA-RNA duplex region of 171 *trans*-NAT pairs (Table 7). Among them, 110 siRNAs could be generated by more than one *trans*-NAT pair. Comparison of siRNA density (matched siRNA number versus sequence length) between the pairing and non-pairing regions of the 171 *trans*-NAT pairs revealed that the siRNA density in duplex regions is 1.75 times higher than that in single-strand regions (14 siRNA per 1,000 nt versus 8 siRNA per 1,000 nt). SiRNAs generated from the duplex region of a *trans*-NAT pair could anneal to the antisense transcript and prime the synthesis of double-strand RNAs through RNA-dependent RNA polymerase (RDRP), thereby generating more siRNAs from sequences 5' to the original duplex region. For this reason, only sequences from the 3' end of the duplex region to the 3' end of the transcript that could not produce RDRP-generated siRNAs were considered in the siRNA density analysis.

Expression profile comparison of the *trans*-NAT specific siRNAs between the *Arabidopsis *wild-type and RNA-dependent RNA polymerase 2 (*rdr2*) loss-of-function mutant [31] showed that, out of the 148 siRNAs, only 1 was found in the *rdr2 *mutant. This result suggests that at least some siRNAs generated by *trans*-NATs are RDR2-dependent.

Because a large proportion of the 171 siRNA-related *trans*-NAT pairs were formed by putative transcripts from genes annotated as encoding hypothetical proteins, we asked whether some of these genes are uncharacterized transposable elements. To address this question, we extracted the corresponding genomic regions of genes involved in the 171 *trans*-NAT pairs, and used RepeatMasker to examine the homology of these sequences with known transposable elements. The results showed that 101 *trans*-NAT pairs had at least one transcript whose corresponding genomic region displayed high homology to transposable elements listed in the Repbase, indicating that these genes might be derived from transposons.

### *Trans*-NATs and alternative splicing

Another reported function of *trans*-NATs is to alter the splicing pattern of their corresponding sense transcripts by base pairing, thereby masking certain splicing sites [10,11]. To explore the potential roles of *Arabidopsis trans*-NATs in regulating alternative splicing, we compared the proportion of genes with alternative splicing in our predicted *trans*-NAT pairs with that of all genes in the *Arabidopsis *genome. A previous study using full-length cDNAs showed that about 11.59% of *Arabidopsis *transcription units had alternative splicing events [32]. For the 658 predicted *trans*-NAT pairs that had corresponding annotated genes for both transcripts, 127 pairs had one transcript with known alternatively spliced gene products, and another 3 pairs had alternatively spliced forms for both transcripts (Table 8). These data show that *Arabidopsis trans*-NAT pairs have a much higher proportion of alternative splicing events (19.76%) compared to all transcription units in the genome (11.59%), suggesting that some *trans*-NATs might function in regulating alternative splicing in *Arabidopsis*. Furthermore, among these 130 *trans*-NAT pairs, about 60% had antisense pairing regions overlapped with alternatively spliced exons, suggesting that the binding of antisense transcripts to the pre-mRNA of their sense partners could cause the exclusion of the pairing region from the resulting mature sense mRNAs.

## Discussion

As a newly identified regulatory mechanism of gene expression in eukaryotes, antisense regulation has attracted increasing attention in recent years. Here we provide the first genome-wide *trans*-NAT prediction results in plants with the identification of 1,320 putative *trans*-NAT pairs in *A. thaliana*. The potential roles of *trans*-NATs in regulating alternative splicing and gene silencing were also explored.

Although a large amount of *cis*-NATs has been identified in most model organisms experimentally or computationally during the past few years [1-7], little attention has been paid to *trans*-NATs. The widespread existence of *trans*-NATs was noted in a recent attempt to identify double-stranded RNA molecules in human normal mammary epithelial and breast cancer cell lines [21]. In that experiment, about 50% of the cloned double-stranded RNAs were derived from *trans*-NAT pairs.

NAT pairs are transcripts with sequence complementarity to each other; however, there is no clear-cut criterion to define what the degree of complementarity should be for the two transcripts to form *trans*-NATs. Unlike *cis*-NATs, which can be easily identified by comparing the genomic loci of two transcripts, *trans*-NATs are more difficult to identify and, therefore, provide a greater computational challenge. Here, we chose a relatively strict criterion to define *trans*-NATs. Only transcript pairs with a sequence complementary region longer than 100 nt or that covers more than 50% of the length of the shorter transcript were considered as *trans*-NAT pairs. It is possible that there could be other transcript pairs with shorter sequence complementary regions that did not fit into our criterion but could also function as *trans*-NATs.

The RNA hybridization program showed that, for most *trans*-NAT pairs, their *in silico *lowest energy annealing forms contain long double-stranded RNA regions, as we predicted. However, unlike *cis*-NATs, which may function at the transcription level, two transcripts of a *trans*-NAT pair must interact physically to regulate each other. Using tissue specific gene expression data from the public MPSS database and the *in situ *hybridization data of *Arabidopsis *root cells, we were able to demonstrate that the two transcripts of most *trans*-NAT pairs with available data are expressed in the same tissue under certain conditions and in the same root cells, suggesting they have the potential to interact *in vivo*.

Phylogenetic analysis showed that the orthologs in poplar or rice of one transcript of about 50% of *Arabidopsis trans*-NAT pairs also had *trans*-NAT partners. However, we found only one *Arabidopsis trans*-NAT pair with both transcripts and pairing relationship conserved in poplar and rice. For other *Arabidopsis trans*-NAT pairs in which both transcripts retained the *trans*-NAT relationship in poplar or rice, homologs of both the sense and antisense transcripts of *Arabidopsis *had recruited their own *trans*-NAT partners. This result suggests that antisense regulation may be important for only one transcript of a *trans*-NAT pair. The lack of phylogenetic conservation of some *trans*-NAT pairs also indicates that antisense regulation might have some species-specific functions.

The interlaced relationships between some *cis*- and *trans*-NAT pairs suggest that antisense transcripts could form complex regulatory networks in eukaryotes. As illustrated by the case of transcripts of UDP-glucosyl transferase gene members, one antisense transcript could regulate many UDP-glucosyl transferase transcripts in either a *cis*- or *trans*-manner, suggesting the existence of co-regulation of these UDP-glucosyl transferase transcripts by the same signaling pathway. The high homology of these transcripts at the sequence level also indicates that they might have similar biological functions. On the other hand, several antisense transcripts could also form *trans*-NAT pairs with the same UDP-glucosyl transferase transcript. This result suggests that the latter might respond to several signals, each regulating the expression of a different antisense transcript. Complex regulation amongst UDP-glucosyl transferase transcripts may also occur as some transcripts could form both *cis*- and *trans-*NAT pairs. We noted that some microRNA targets were also included in either the *cis*- or *trans*-NAT list, or both. For example, transcripts of the *NAC1 *gene (At1g56010), which is a target of microRNA ath-Mir164 [33], have both *cis*- and *trans*-NATs. This finding suggests that gene expression regulation at the RNA level could form complex networks in eukaryotes. One gene or its product might be regulated by one mechanism under one condition, whilst other mechanisms may operate under other conditions. The recently identified siRNAs from one *Arabidopsis cis*-NAT pair under high salt conditions has also raised such possibility [34].

The siRNAs identified from the double-strand region of some *trans*-NAT pairs suggested a potential role of *trans*-NATs in inducing RNA silencing. However, this hypothesis should be questioned by the fact that the number of *trans*-NAT associated siRNAs does not differ significantly from those of other transcripts. One possible explanation for this discrepancy could be that, like most other gene regulatory mechanisms, antisense regulation also has tissue or temporal specificity, or could only be induced under specific conditions, such as abiotic or biotic stresses. Thus, it would be difficult to identify *trans*-NAT derived siRNAs by a general small RNA cloning method. The observation that, in *Arabidopsis*, some *cis*-NAT generated siRNAs can only be detected under high salt conditions [34] provides some support for this hypothesis. Another reason could be that inducing RNA silencing is the function of only a small proportion of *trans*-NAT pairs, whilst many *trans*-NAT pairs may function in other regulatory pathways as discussed below. The third possibility is that, notwithstanding the sequence complementarity, the two transcripts of a *trans*-NAT pair are not related and rarely form RNA-RNA duplexes within the cell. However, given the large amount of *trans*-NAT-related double-stranded RNA duplexes cloned from human, this possibility seems to be remote [21].

The study of the relationship of *trans*-NATs and alternative splicing revealed that alternative splicing events occurred about two times more frequently in *trans*-NAT pairs compared to all transcripts in the genome (Table 8), suggesting that some *trans*-NATs might function by regulating the splicing pattern of their sense partners. The overlapping of pairing regions of some *trans*-NAT pairs with alternatively spliced exons further supports the above hypothesis. Since alternative splicing has not been investigated in transcripts of full-length cDNAs without an annotated gene match, to ensure a fair comparison, only *trans*-NAT pairs in which both transcripts have corresponding annotated genes were included in our analysis.

*Trans*-NATs may also function by repressing translation to reduce the amount of proteins produced by the sense transcript, inducing RNA editing, thereby changing the primary amino acid sequence of a protein, masking certain regions of the sense transcript to block the access of regulatory RNA binding proteins, or causing structural changes of the sense transcript to alter its biological functions. All these possibilities need to be tested experimentally in the future.

## Conclusion

Together with previous reports on *cis*-NATs [7], we have now completed antisense prediction work in *Arabidopsis *by identifying 1,320 *trans*-NAT pairs. Our results show that antisense transcripts are more widespread in plants than hitherto recognized. The putative *trans*-NAT pairs reported here will serve as a data resource for biologists to investigate the function of *trans*-NATs. The complex networks formed by antisense transcripts are important for deciphering gene expression regulatory networks of plants at the RNA level.

## Materials and methods

### Sequence resources and transcript clusters

The sequences and genomic coordinates of 28,952 annotated *A. thaliana *genes was obtained from TIGR (release version 5) [35]. The *Arabidopsis *full-length cDNA sequences used in this study were collected from UniGene and RIKEN datasets. The *Arabidopsis *UniGene dataset (Build#48) was downloaded from the National Center for Biotechnology Information (NCBI) UniGene Resources [36]. A total of 20,687 full-length cDNA sequences were extracted from the *Arabidopsis *UniGene dataset by selecting sequences marked as 'Full-length cDNA'. The RIKEN Arabidopsis full-length cDNA dataset, which contains 13,181 sequences, was downloaded from the RIKEN Arabidopsis Genome Encyclopedia [37].

Full-length cDNA sequences were aligned to the *Arabidopsis *genome by the BLAT program [38]. Sequences with unique genomic location and at least 95% identity to the genome were used in this analysis. Full-length cDNAs and annotated genes derived from the same genomic locus (≥ 90% sequence coverage) were grouped into one transcript cluster.

Annotated gene sequences and full-length cDNAs of *Oryza sativa *were downloaded from TIGR [35] and NCBI UniGene resources [36], respectively. Annotated gene sequences of *Populus trichocarpa *were downloaded from DOE Joint Genome Institute [39]. Both poplar and rice sequences were clustered in the same way as described for *Arabidopsis*.

### Prediction of *trans*-NAT pairs

*Trans*-NAT pairs were identified by aligning transcript clusters to themselves to search for transcript pairs with high sequence complementarity to each other. In this study, we used the following criteria to define *trans*-NATs. For two transcripts with different genomic origins, if all paired regions between them cover more than half of the length of either transcript, the two transcripts were considered as a valid *trans*-NAT pair and referred to as a 'high-coverage' *trans*-NAT pair. Otherwise, if two transcripts have a continuous paring region with a length longer than 100 nt, they are classified as '100 nt' *trans*-NAT pairs. *Cis*-NAT pairs and pairs including transponsons or pesudogenes were removed from each category. Double-stranded RNA duplexes formed by the same sense transcript with alternatively spliced antisense transcripts from the same gene were considered as separate pairs if the pairing patterns between the sense and antisense transcripts were different.

### Structural analysis of *trans*-NAT pairs

The melting profile of two RNA molecules of a *trans*-NAT pair was predicted using the hybrid software [23,24]. Wecompared the total pairing regions from the results provided by the hybrid software with those from the BLAST software of each *trans*-NAT pair. If at least 80% of the BLAST results-based pairing regions of one transcript in a *trans*-NAT pair were also predicted as pairing regions by the hybrid software, we considered our prediction to be consistent with the results from the hybrid software.

### Expression evidence for *trans*-NAT pairs

The *Arabidopsis *MPSS expression data were downloaded from the public *Arabidopsis *MPSS database at the University of Delaware [40]. The MPSS data contained 297,313 17-nt and 263,552 20-nt signature sequences of *Arabidopsis *transcripts from 17 tissues or plants under different treatments. Only MPSS sequences with 'reliable' (present in more than one sequencing run) and 'significant' (TPM ≥ 4) expression patterns and that have unique genomic loci were used in this study. Normalized abundance (TPM) refers to the transcript abundance (Parts Per Million) obtained from the sequencing procedure. There were 82,885 17-nt tags and 81,586 20-nt tags that satisfied the above criteria.

*In situ *hybridization data of *Arabidopsis *root cells were downloaded from AREX [25]. Two transcripts of a *trans*-NAT pair were considered to be co-expressed if they were detected in the same cell.

### Phylogenetic conservation of *trans*-NAT pairs

Protein sequences derived from transcripts involved in *Arabidopsis*, rice and poplar *trans*-NAT pairs were compared using the BLASTP program. High similarity pairs with an E-value less than 10^-30 ^and alignment coverage greater than 50% of query sequence were considered as homologous sequences.

### Small RNA matches of *trans*-NAT pairs

The small RNA data used in this analysis were obtained from the *Arabidopsis *MPSS database [30,31,40] These small RNA sequences were aligned to all transcript clusters forming *trans*-NAT pairs to search for *trans*-NAT originated small RNAs. Small RNAs that could be mapped to the pairing region of *trans*-NAT pairs were considered as *trans*-NAT induced siRNAs.

### Transposable element prediction

Transcripts of *trans*-NAT pairs with siRNA matches were first mapped to the *Arabidopsis *genome using the BLAT program [38]. The corresponding genomic regions were extracted and screened by RepeatMasker [41] Genomic sequences with high sequence homology to transposable elements collected in the Repbase (RepeatMasker score was greater than 250 and homology region was longer than 40% of the entire sequence length) were considered to be transposon-like sequences.

## Additional data files

The following additional data are available with the online version of this paper. Additional data file 1 contains the list of predicted *Arabidopsis trans*-NAT pairs and results of their analysis. Additional data file 2 provides *in situ *hybridization data obtained from the AREX database for some *Arabidopsis trans*-NAT pairs. Additional data file 3 shows the phylogenetic tree of UDP-glucosyl transferase family proteins involved in antisense pairs.

## Supplementary Material

Additional data file 1 Predicted *Arabidopsis trans*-NAT pairs and results of their analysis Click here for file

Additional data file 2 *In situ *hybridization data obtained from the AREX database for some *Arabidopsis trans*-NAT pairs Click here for file

Additional data file 3 Phylogenetic tree of UDP-glucosyl transferase family proteins involved in antisense pairs Click here for file

## Figures and Tables

**Table 1:** Summary of *trans*-NAT pairs and their corresponding full-length cDNAs

		No. of *trans*-NAT pairs		
		
*Trans*-NAT groups	Total *trans*-NAT pairs	Both transcripts with FL-cDNA	One transcript with FL-cDNA	No matching FL-cDNA
High-coverage	368	162	117	89
100 nt	952	496	327	129
Total	1,320	658	444	218

FL-cDNAs, full-length cDNAs.

**Table 2:** Expression analysis of *trans*-NAT pairs using MPSS data

		No. of *trans*-NAT pairs			
			
*Trans*-NAT groups		Without MPSS tag	Single strand with MPSS tag	Both strands with MPSS tag (same tissue)	No. of total pairs
HC	17 nt MPSS tag	196	125	47 (37)	368
	20 nt MPSS tag	197	115	56 (40)	
	Either 17 nt or 20 nt MPSS tag	192	118	58 (50)	
100 nt	17 nt MPSS tag	276	436	240 (184)	952
	20 nt MPSS tag	269	428	255 (199)	
	Either 17 nt or 20 nt MPSS tag	252	430	270 (231)	

Data in parentheses are number of *trans*-NAT pairs with expression in the same tissue. HC and 100-nt refer to the 'high-coverage' and the '100 nt' *trans*-NAT pair categories, respectively.

**Table 3:** Tissue specific MPSS data demonstrate co-expression pattern of some *trans*-NAT pairs

	Libraries																
	
ID	CAF	INF	LEF	ROF	SIF	API	AP3	AGM	INS	ROS	SAP	SO4	S52	LES	GSE	CAS	SIS
Pair A																	
At1g50020	0	18	6	1	0	12	22	1	17	0	0	0	0	0	73	0	0
At1g04820	0	0	0	0	0	19	13	8	13	1	3	0	0	0	0	0	0
Pair B																	
At5g02370	0	0	0	0	0	36	15	20	16	0	56	1	19	29	21	28	39
At3g09390	0	0	0	0	0	60	36	27	14	0	55	8	4	28	362	0	0

The MPSS data of each transcript within each tissue or differently treated plants are shown to reflect their expression levels

**Table 4:** Co-expression analysis of *trans*-NAT pairs using *Arabidopsis *root cell *in situ *hybridization results

	No. of *trans*-NAT pairs		
	
*Trans*-NAT groups	Both transcripts with *in situ *data	One transcript with *in situ *data	No *in situ *data
High-coverage	35 (25)	66	32
100 nt	320 (212)	169	36
Total	355 (237)	235	68

Numbers of *trans*-NAT pairs with expression difference between sense and antisense transcripts less than two-fold according to the *in situ *hybridization data are shown in parentheses. *Expression difference ≤ 2-fold.

**Table 5:** Over-represented gene families or functional groups in *Arabidopsis trans*-NAT pairs

Rank	N	X	P-adj	GO attribute
1	43	167	<0.001	0008194: UDP-glycosyl transferase activity
2	197	2,634	<0.001	0016757: transferase activity
3	16	40	<0.001	0016168: chlorophyll binding
4	102	1,158	<0.001	0005515: protein binding
5	16	58	<0.001	0042546: cell wall biosynthesis
6	10	25	<0.001	0030076: light-harvesting complex
7	25	195	0.003	0006511: ubiquitin-dependent proteolysis
8	42	437	0.003	0006464: protein modification
9	53	611	0.003	0007165: signal transduction
10	392	7,058	0.006	0003824: catalytic activity
11	23	194	0.013	0009733: response to auxin stimulus

N, number of transcripts from the same GO category involved in *Arabidopsis trans*-NAT pairs; P-adj, adjusted p value calculated by 1,000 null-hypothesis simulations using Fisher exact test; X, number of genes from the same GO category in *Arabidopsis *genome.

**Table 6:** Phylogenetic conservation of *Arabidopsis trans*-NAT pairs

	No. of *trans*-NAT pairs		
	
	Conserved in both poplar and rice	Conserved in poplar only	Conserved in rice only
*Trans*-NAT pattern conserved for both transcripts in the same pair	1	0	0
*Trans*-NAT pattern conserved for both transcripts	13	14	58
*Trans*-NAT pattern conserved for single transcript	320	134	229

**Table 7:** siRNA matches on *trans*-NAT pairs

	No. of pairs carrying siRNAs on overlapping region	No. of siRNAs matching on overlapping region
HC	106	98
100 nt	65	77
Total	171	148

HC and 100 nt refer to the 'high-coverage' and the '100 nt' *trans*-NAT categories, respectively.

**Table 8:** Number of genes with alternative splicing in the *Arabidopsis *genome and in predicted *trans*-NAT pairs

	No. of TUs/pairs with alternative splicing	Total TUs/pairs	Proportion (percent)
All annotated genes	1,764	15,214	11.59
*Trans*-NAT pairs	130	658	19.76

TU refers to transcription units with multiple sequences.
