# Supplementary material for: Prediction of trans-antisense transcripts in Arabidopsis thaliana
Source: Genome Biol. 2006 Oct 13;7(10):R92. doi: 10.1186/gb-2006-7-10-r92 (PMC1794575; doi:10.1186/gb-2006-7-10-r92)

Additional data file 3. Phylogentic tree of UDP-glucosyl transferase family proteins involved in antisense pairs.

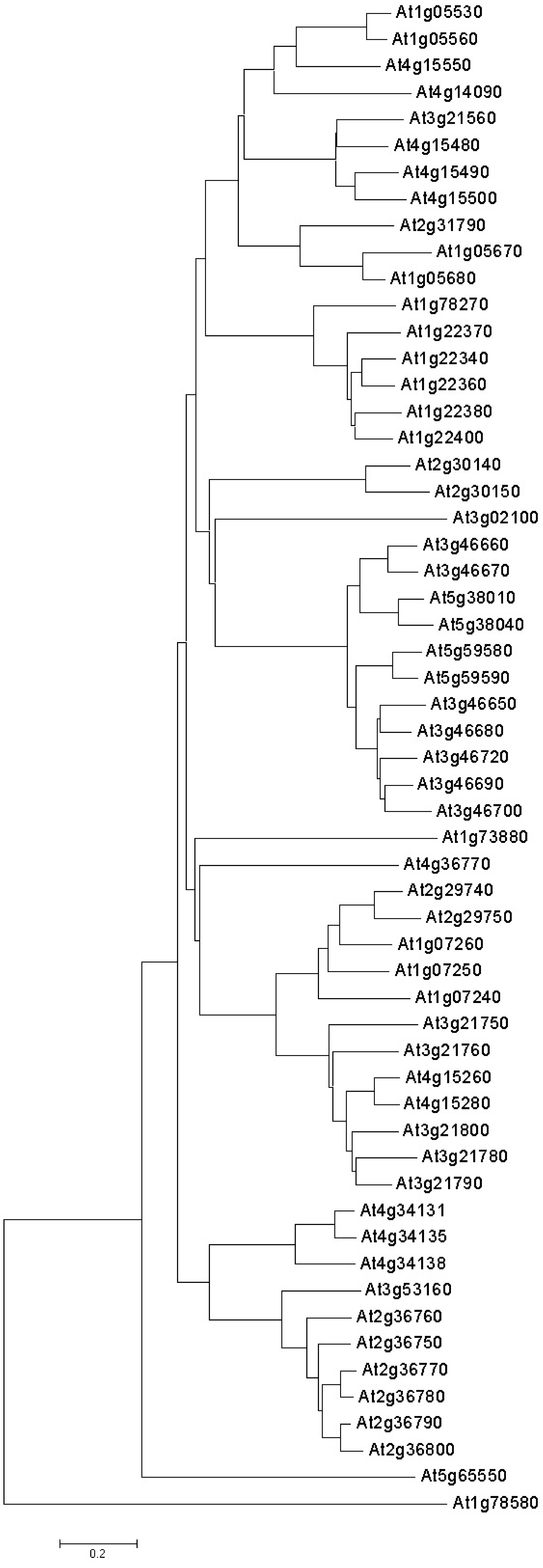

Supplement: Additional data file 3 — Phylogenetic tree of UDP-glucosyl transferase family proteins involved in antisense pairs [file gb-2006-7-10-r92-S3.pdf]
